# Letter to editor – observation on the article titled “Vaccine-Induced Thrombotic Thrombocytopenia (VITT): first report from India”

**DOI:** 10.1186/s12959-023-00470-x

**Published:** 2023-04-24

**Authors:** Jyoti Kotwal, K. V. Vinu Balraam

**Affiliations:** 1 Department of Hematology, Sir Ganga Ram Hospital, Delhi, India; 2 Department of Hematology, All India Institute of Medical Sciences, New Delhi, India

**Keywords:** COVID-19, Thrombosis, VITT

## Abstract

The first case of Vaccine-Induced Thrombotic Thrombocytopenia (VITT) was reported in the letter-to-editor submission in the journal of Indian Journal of Hematology and Blood Transfusion which was published online on 29th Sep 2021. Whereas, an article published in your journal on 04th Mar 2022 has been titled as first report of VITT from India which is a very conflicting statistic. The former article under reference has been diagnosed by a confirmatory functional assay as per the recommended guidelines and is thus genuinely the first case reported in this country.

Dear Editor,

As we all know that COVID-19 has created a grave situation ever since the inception of pandemic in Nov 2019. To curb the situation, the mankind worked tirelessly and produced vaccines with the vision of mass vaccination. However, an extremely rare vaccine associated adverse event was observed in a sparse subpopulation of individuals especially those who received adenoviral vector-based vaccines. This complication was termed as Vaccine-induced Immune Thrombotic Thrombocytopenia (VITT).

In this context, I would like to bring to your notice that the first confirmed case from India was reported by Mishra et al. in his article titled *“COVID-19 Vaccine-Induced Thrombosis and Thrombocytopenia: First Confirmed Case from India”* [1]. The index case was confirmed as per the recommended guidelines of American Society of Hematology using a functional assay i.e. platelet activation test for heparin induced thrombocytopenia and thrombosis (HITT) which is also the recommended testing modality for diagnosing VITT. The article under reference was submitted to the Indian Journal of Hematology and Blood Transfusion on 11th August 2021 and was accepted on 18th September 2021. The same was published online on 29th September 2021.

The article titled *“Vaccine-Induced Thrombotic Thrombocytopenia (VITT): First Report from India”* by John et al. was published in your esteemed journal [2]. This submission was received by your journal on 19th September 2021, which was accepted by your journal on 17th February 2022 and published online on 04th March 2022. Hence, the title of this article and the discussion contained in it doesn’t vindicate the fact that it is actually the first report from India.

You are requested to delve into this matter and make necessary refinements so that the article in reference number 1 gets the due credit and the article printed in your journal looks error-free.

## Data Availability

Letter to editor to highlight an oversight in the publication.
